# Adult stem cells in endometrial regeneration: Molecular insights and clinical applications

**DOI:** 10.1002/mrd.23476

**Published:** 2021-05-20

**Authors:** Qiaoying Lv, Lulu Wang, Xuezhen Luo, Xiaojun Chen

**Affiliations:** ^1^ Department of Gynecology Obstetrics and Gynecology Hospital of Fudan University Shanghai China

**Keywords:** adult stem cells, Asherman syndrome, endometrial cancer, endometrial regeneration

## Abstract

Endometrial damage is an important cause of female reproductive problems, manifested as menstrual abnormalities, infertility, recurrent pregnancy loss, and other complications. These conditions are collectively termed “Asherman syndrome” (AS) and are typically associated with recurrent induced pregnancy terminations, repeated diagnostic curettage and intrauterine infections. Cancer treatment also has unexpected detrimental side effects on endometrial function in survivors independently of ovarian effects. Endometrial stem cells act in the regeneration of the endometrium and in repair through direct differentiation or paracrine effects. Nonendometrial adult stem cells, such as bone marrow‐derived mesenchymal stem cells and umbilical cord‐derived mesenchymal stem cells, with autologous and allogenic applications, can also repair injured endometrial tissue in animal models of AS and in human studies. However, there remains a lack of research on the repair of the damaged endometrium after the reversal of tumors, especially endometrial cancers. Here, we review the biological mechanisms of endometrial regeneration, and research progress and challenges for adult stem cell therapy for damaged endometrium, and discuss the potential applications of their use for endometrial repair after cancer remission, especially in endometrial cancers. Successful application of such cells will improve reproductive parameters in patients with AS or cancer. Significance: The endometrium is the fertile ground for embryos, but damage to the endometrium will greatly impair female fertility. Adult stem cells combined with tissue engineering scaffold materials or not have made great progress in repairing the injured endometrium due to benign lesions. However, due to the lack of research on the repair of the damaged endometrium caused by malignant tumors or tumor therapies, the safety and effectiveness of such stem cell‐based therapies need to be further explored. This review focuses on the molecular insights and clinical application potential of adult stem cells in endometrial regeneration and discusses the possible challenges or difficulties that need to be overcome in stem cell‐based therapies for tumor survivors. The development of adult stem cell‐related new programs will help repair damaged endometrium safely and effectively and meet fertility needs in tumor survivors.

## 1 INTRODUCTION

Recurrent miscarriage, termination of pregnancy, repeated diagnostic curettage, and intrauterine infections can cause irreversible endometrial damage (Polishuk et al., 1975; Schenker & Margalioth,^1982^; Taskin et al., 2000). The trauma to the endometrium often produces partial or complete obliteration of the uterine cavity and/or the cervical canal, which results in menstrual abnormalities, infertility, recurrent pregnancy loss, and other pregnancy complications (Yu et al., 2008). This condition is called “Asherman syndrome” (AS) or “Intrauterine adhesions.” Studies have shown that 68% of patients with AS have menstrual abnormalities, including hypomenorrhea and even amenorrhea caused by cervical adhesion‐induced menstrual flow obstruction or severe endometrial fibrosis, and 43% of such patients are diagnosed with infertility (Schenker & Margalioth, 1982). Patients with a milder degree of adhesions can become pregnant but are always at risk of repeated pregnancy loss because of defective normal endometrial tissue and vascularization of the residual endometrial tissue to support implantation (Polishuk et al.,^1975^) In terms of obstetric complications, patients with AS consistently demonstrated an increased risk of preterm delivery, placenta accrete and subsequent postpartum hemorrhage (Schenker & Margalioth,^1982^; Valle & Sciarra,^1988^). A retrospective study reported that patients with AS undergoing hysteroscopic management showed a 79% pregnancy rate and a 63.7% live birth rate, but there was still a 23.4% miscarriage rate, a 17.6% rate of abnormal placentation, a 4.7% rate of postpartum hysterectomy, and a 29.4% rate of premature delivery (Deans et al., 2018). Pathologically, AS is a condition in which the endometrial stroma is largely replaced by fibrous tissue, and the glands usually comprise inactive cuboidal/columnar epithelium, and even do not respond to hormones (Al‐Inany, 2001). Repairing such damaged endometrium is currently a difficult clinical and scientific problem.

The human endometrium is a highly regenerative tissue that undergoes periodic proliferation, differentiation, and shedding in women of childbearing age. In the past two decades, studies have identified a small number of endometrial stem/progenitor cells in the basal and functional layers of the endometrium (Gargett et al., 2016). Repeated diagnostic curettage, abortions, or infections often damage these endometrial stem/progenitor cells (Gargett et al., 2016). Although such cells can survive without estrogen, their stem cell niches require estrogen to activate them (Gargett et al., 2012). Currently, adult stem cells derived from various tissues have shown great potential in the repair of injured endometrium and some studies have entered the clinical trials (Cao et al., 2018; Xu et al., 2017). Successful applications of adult stem cells to treat extensive intrauterine adhesions, endometrial atrophy (EA) and scarring might greatly improve menstruation and reprogram female fertility.

In addition, because cancer treatment brings unexpected side effects on the reproductive system, oncofertility has become an emerging field of medicine and research. Increasing evidence has shown that chemotherapy and radiotherapy for cancer have off‐target effects on uterine function in female cancer survivors independently of ovarian effects (Griffiths et al., 2020). Clinical studies have demonstrated that women who have received chemotherapy have lower pregnancy rates and their offspring have reduced live birth weights compared with sibling controls (Chow et al., 2016). Similarly, a nested cohort study demonstrated that female survivors with childhood cancers exposed to radiotherapy were at increased risk of pregnancy complications, preterm delivery, and low birth weight infants (van de Loo et al., 2019). In terms of etiology, reports showed that cancer therapies can cause reductions in uterine volume and elasticity, and endometrial atrophy and insufficiency (Teh et al., 2014). Mechanistically, it has been speculated that endometrial stem/progenitor cells and their niche cells can be damaged in a similar way, just as chemotherapy and radiotherapy can induce intestinal stem cell apoptosis and affect the gastrointestinal stem cell niches (Hu et al., 2016; Zhan et al., 2016). But there is currently no direct evidence to support this concept in the endometrium. In the early stage of endometrial cancer, the invasive effects on the basal layer, and fertility‐preserving regimens, such as high‐dose progesterone and repeated diagnostic intrauterine operations, are more likely to cause endometrial thinning, reduce the implantation rate, and require more embryo transfer cycles and good‐quality embryos for achieving a live‐birth in assisted reproductive technology programs when compared to control women without cancers (Fujimoto et al., 2014). Therefore, recovering normal endometrial function after tumor remission, especially in patients with endometrial cancers, is also an important challenging area.

There have been many basic and clinical studies on adult stem cells from different tissues to repair the injured endometrium. Here, we review the biological mechanisms of endometrial repair, research progress in adult stem cell therapy for damaged endometrium, and discuss the potential applications of the use of such cells in endometrial repair after cancer remission, especially for endometrial cancer.

## 2 METHODS

Papers referenced in this review were retrieved from PubMed with the search terms “Asherman syndrome,” “adult stem cells,” OR “endometrial regeneration” up to March 2020. The literature review included animal models and human studies and related molecular mechanisms.

## 3 BIOLOGICAL MECHANISMS OF ENDOMETRIAL REPAIR

The human endometrium is a highly dynamic tissue that, when exposed to ovarian estrogen and progesterone, undergoes periodic shedding, repair, regeneration, and remodeling, and prepares for embryo implantation (Evans et al., 2016). Understanding the cyclic physiological and biological processes of normal human endometrium is essential to treat abnormal endometrial lesions and repair injured endometrial tissue caused by factors such as repeated intrauterine operations, infections, and cancer therapy.

### 3.1 Endometrium

The human endometrium is divided into functional and basal layers anatomically and functionally. The functional layer derived from the basal layer is the “fertile ground” for embryo implantation. The endometrium of this functional layer is regulated by ovarian hormones and undergoes periodic proliferative and secretory changes. If the oocyte is not fertilized, this layer then undergoes ischemic necrosis and shedding, which results in menstruation caused by the regression of ovarian luteum functions as well as estrogen and progesterone withdrawal. Conversely, the endometrium of the basal layer is close to the myometrium and remains unaffected by the cyclic changes of ovarian hormones (Evans et al., 2016). It regenerates and repairs the endometrial wound after menstruation and then rebuilds the functional layer.

### 3.2 Molecular mechanisms of endometrial tissue destruction and regenerative repair

Menstruation is initiated after ovarian hormone withdrawal, which is mediated by complex endocrine and paracrine signaling in the local endometrium. Interestingly, endometrial tissue destruction and re‐epithelialization occur simultaneously. Re‐epithelialization begins at approximately 36 h after menstruation onset, which requires approximately 48 h to complete (Garry et al., 2009). Hysteroscopic analysis of the endometrium in the menstrual period has demonstrated that endometrial resolution is a zonal event, that is, areas adjacent to the shedding endometrium have intact endometrial tissue from the previous menstrual cycle and there are areas that have completed re‐epithelialization (Garry et al., 2009). Endometrial stromal cells are decidualized upon exposure to progesterone; decidualized stromal cells (DSCs) express the progesterone receptor (PR) during the premenstrual period and detect progesterone withdrawal, supporting a role of DSCs in responding to endocrine signals and transmitting paracrine signals during menstruation (Evans & Salamonsen, 2014; Mote et al., 2000). In vitro experiments have shown that progesterone withdrawal inhibits the expression of superoxide dismutase in DSCs and promotes free oxygen production. The latter upregulates nuclear factor‐κB and cyclooxygenase‐2 and the expression of inflammatory factors such as prostaglandin F2α, and chemotaxis factors, and then recruits inflammatory cells such as macrophages, eosinophils, and neutrophils to the menstrual endometrium (Evans & Salamonsen,^2014^; Sugino et al., 2004). These inflammatory cells secrete proteolytic enzymes that contribute to the destruction of endometrial tissues while promoting the expression of proteases and gene products related to extracellular matrix synthesis and repair, which initiate endometrial repair. Those factors, including activin, vascular endothelial growth factor (VEGF), cysteine‐rich secretory protein 3, and galectin‐7, and development‐related pathways such as the Wnt pathway and mesenchymal‐epithelial transition, promote endometrial re‐epithelization and regeneration after menstruation but do not rely on estrogen (Evans et al., 2014, 2015; Fan et al., 2012). When the endometrium is re‐epithelialized completely, estrogen is needed to stimulate endometrial epithelial and stromal cell proliferation.

## 4 ENDOMETRIAL STEM/PROGENITOR CELLS

**Figure 1:**
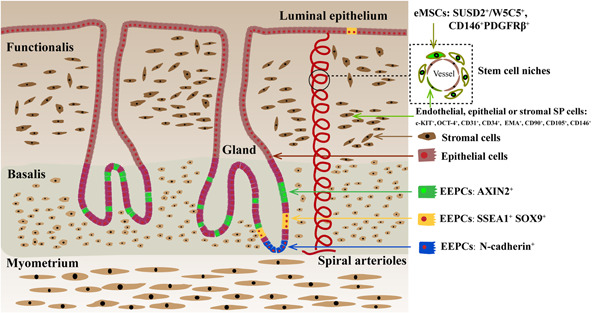
Human endometrial stem cells Human endometrial stem cells (endoSCs), including endometrial epithelial progenitor cells (EEPCs), endometrial mesenchymal stem cells (eMSCs), and side population (SP) cells, have been identified in the endometrium. EEPCs are mainly located in glands of the basal layer of the endometrium. Among the stem markers in EEPCs, AXIN2 marks most epithelial cells in the basalis, N‐cadherin only the bases of the glands adjacent to the myometrium, SSEA1 and SOX9 are co‐localized and they are in the basalis (and luminal epithelium in the functionalis) and proximal to the N‐cadherin+ cells with little overlap. eMSCs are located around blood vessels in the endometrium of functional and basal layers, and exist in shedding menstrual blood, which express stem markers such as SUSD2/W5C5, CD146, and PDGFRβ. Endothelial, epithelial, and stromal SP cells have the ability to exclude the DNA‐binding dye Hoechst 33342 through the ATP‐binding cassette transporter, which express multiple types of cell markers, including high levels of undifferentiated cell markers c‐KIT and OCT‐4, endothelial cell markers CD31 and CD34, epithelial cell marker EMA, and mesenchymal stem cell marker CD90, CD105, and CD146. EndoSCs are involved in endometrial tissue regeneration. Near the rapidly growing spiral arterioles of the human endometrium, there is an area around the blood vessels, which promotes the self‐renewal of stem cells. This area has the function of a stem cell niche, which promotes endometrial cyclic regeneration. This diagram was adapted from Hum Reprod Update 2016; 22: 137‐163

Studies have suggested that endometrial stem cells (EndoSCs) in the endometrial tissue can participate in endometrial regeneration and repair (Chan et al., 2004; Gargett & Masuda, 2010; Santamaria et al., 2018). Currently, researchers have identified a small number of EndoSCs with a colony‐forming ability, self‐renewal, and differentiation potential in endometrial tissue, such as endometrial epithelial progenitor cells (EEPCs), mesenchymal stem cells (eMSCs), and side population (SP) cells as shown in Figure 1. There are several markers expressed by human EEPCs and eMSCs that purify these cells for which adult stem cell activity has been reported and is described in the following paragraphs. However, it is unclear whether one or more EndoSC types are involved in endometrial tissue regeneration.

### 4.1 EEPCs

EEPCs have been identified in glands of the basal layer of the endometrium, which begin to re‐epithelialize within 48 h of menstrual initiation (Evans et al., 2016). Gargett et al. reported that freshly isolated epithelial cells could be used to obtain epithelial cell colony‐forming cells/units (CFUs) at a frequency of 1/174 (Gargett et al., 2009). Moreover, large epithelial CFUs have high proliferative potential, can produce billions of cells, and undergo unilineage differentiation into cytokeratin (CK)^+^ gland‐like organoids in three‐dimensional culture. Currently, there is increasing interest in the reports on stem cell markers of EEPCs. Valentijn et al. found that stage‐specific embryonic antigen‐1 (SSEA‐1) is highly expressed in the endometrial basal layer of premenopausal and postmenopausal women, and SSEA‐1^+^ epithelial cells have higher telomerase activity, lower expression of ERα and PR, and generate more gland‐like structures in three‐dimensional culture conditions compared with SSEA‐1−epithelial cells (Valentijn et al., 2013). The classical Wnt/β‐catenin pathway is an important regulator for stem cell maintenance and differentiation (Waghmare & Page‐McCaw, 2018). SOX9 and β‐catenin in this pathway are highly expressed in SSEA‐1^+^ epithelial cells, which further suggests that SSEA‐1 can be used to identify EEPCs (Valentijn et al., 2013). Nguyen et al. conducted in vitro stem cell assays to confirm that N‐cadherin can also be used to identify EEPCs, although N‐cadherin^+^ epithelial cells express ERα and express less SSEA‐1 and SOX9 (Nguyen et al., 2017). In addition, AXIN2, a Wnt signaling molecule, was described as a human endometrial epithelial basal cell marker (Nguyen et al., 2012). In 2020, Syed et al. demonstrated that endometrial Axin2^+^ cells can fuel endometrial epithelial growth and regeneration and that Axin2 gene mutations drive endometrial carcinogenesis in the murine model (Syed et al., 2020). These findings suggested that stem cell markers in EEPCs with early genetic alterations may lead to the emergence of endometrial cancer stem cells and that these markers provide a means for identifying cells that could be targeted for new therapies and repair of endometrial tissue.

Investigators have also identified label‐retaining cells (LRCs) with quiescent or slow‐cycling phenotype as progenitor cells in the murine endometrium (Chan & Gargett, 2006). Using the principle of label retention, bromodeoxyuridine (BrdU) is injected into newborn mice, and over time cells that are static or dividing slowly, called LRCs, retain BrdU significantly longer than cells that divide more rapidly (Cervello et al., 2007). These LRCs express stem cell factor receptor c‐Kit (CD117), OCT‐4, and other stem cell markers. Although mouse endometrial LRCs do not express ERα, surrounding niche cells expressing ERα transmit estrogen signaling to these EEPCs through the production of epithelial growth factor (EGF), transforming growth factor α (TGFα), and fibroblast growth factor 2 (FGF2), which promote endometrial regeneration (Chan & Gargett, 2006; Janzen, Rosales, et al., 2013).

### 4.2 eMSCs

There are also a small number of eMSCs located around blood vessels in the functional and basal layers of the endometrium, which can also be found in menstrual blood (Gargett et al., 2016). Gargett et al.  found that the CD146^+^PDGFRβ (also known as CD140b)^+^ cell population enriched with colony‐forming cells can differentiate into adipocytes, osteoblasts, fibroblasts, and chondrocytes compared with CD146^‐^PDGFRβ^+^ endometrial stromal cells (Schwab & Gargett, 2007; Gargett et al., 2016). Furthermore, they selected a series of perivascular molecular markers to successfully identify sushi domain containing‐2 (SUSD2, also known as W5C5) as a marker to isolate eMSCs (Masuda et al., 2012). The cloning ability of SUSD2^+^CD146^+^ eMSCs was significantly better than that of CD146^+^PDGFRβ^+^ eMSCs. Compared with SUSD2 antibody‐mediated flow cytometric sorting, SUSD2 antibody‐labeled magnetic bead purification results in less damage to eMSCs and a higher eMSC yield, reflecting the practicality and advantage of magnetic bead sorting of SUSD2^+^ eMSCs (Masuda et al., 2012).

In addition, most of the SUSD2^+^ eMSCs population comprises CD90^+^ (93.3%) perivascular cells, and the colony‐forming ability of CD90^high^ eMSCs is significantly stronger than that of CD90^low^ eMSCs (Schwab et al., 2008). Unlike endometrial stromal cells, eMSCs do not express the ERα protein (Ulrich et al., 2014). Compared with CD146^‐^PDGFRβ^+^ endometrial stromal cells, clonogenic perivascular eMSCs overexpress genes mainly involved in angiogenesis, steroid hormone actions, hypoxic responses, inflammation, and immune regulation, and show activated Notch, TGFβ, insulin‐like growth factor (IGF), and Hedgehog coupled with the G protein‐coupled receptor signaling pathway (Spitzer et al., 2012). Thus, SUSD2^+^ and CD146^+^PDGFRβ^+^ eMSCs play important roles in stromal cell regeneration, angiogenesis at the maternal‐fetal interface, and in immunoregulation (Murakami et al., 2014).

### 4.3 SP cells

According to the ability of adult undifferentiated cells and stem cells to exclude the DNA‐binding dye Hoechst 33342 through the ATP‐binding cassette transporter, low‐level Hoechst 33342‐positive endometrial cells are identified as side population (SP) cells. It has been reported that SP cells express multiple types of cell markers, including high levels of the undifferentiated cell markers c‐KIT and OCT‐4, endothelial cell markers CD31 and CD34, epithelial cell marker EMA, and mesenchymal stem cell markers CD90, CD105, and CD146 (Tsuji et al., 2008). SP cells have a medium telomerase length and the abilities to clone, proliferate, and differentiate into adipocytes and osteoblasts under hypoxic conditions (Cervelló et al., 2010, 2011). Similar to SUSD2^+^ eMSCs, SP cells do not express ERα or PR, but express ERβ as the SP population is predominantly endothelial cells (Masuda et al., 2010). By tracing endometrial SP cells, it was found that they can differentiate into endometrial epithelial cells, endometrial stromal cells, and endothelial cells in vitro, and endometrial SP cells implanted under the kidney capsule differentiate into gland‐like structures. Notably, endothelial cells differentiated from SP cells migrate into the mouse kidney parenchyma and form mature blood vessels, reflecting angiogenesis and endometrial regeneration abilities (Masuda et al., 2010; Miyazaki et al., 2012). SP cells display the genotype, phenotype, and functional characteristics of adult stem cells, which are the stem or progenitor cells of the endometrium.

## 5 ENDOMETRIAL STEM CELL NICHES

The term “stem cell niche” was first proposed by Schofield in 1978 to explain the diversity of self‐renewal of highly purified hematopoietic stem cells after transplantation in mice (Schofield, 1978). It was believed that the self‐renewal ability of stem cells depended on the microenvironment provided by the neighboring cells of the stem cells. Stem cell niches, the specific microenvironment in which stem cells are located, are complex, heterotypic, and dynamically changing structures including different cellular components (e.g., stem and differentiated cells), secreted factors (e.g., chemokines, hormones, and Wnt), extracellular matrix (e.g., collagen fibers and fibronectin), physical parameters (e.g., shear stress and tissue stiffness), environmental signals (e.g., hypoxia and metabolism), and bidirectional regulation of stem cells and their microenvironment (Lane et al., 2014). Near the rapidly growing spiral arteries in the human endometrium, there is an area around them that promotes self‐renewal of stem cells. This area has the function of a stem cell niche, which promotes endometrial cyclic regeneration by maintaining the self‐renewal and clonality for EndoSCs and other non‐endoSCs (Cervello et al., 2007, 2017).

In 2007, stem cells were identified in the small intestine and colon using the marker gene *Lgr5* through lineage‐tracing experiments (Barker et al., 2007). Whether LGR5 is a human endometrial stem cell marker needs to be evaluated using stem cell‐related assays. The expression of this gene can be detected by quantitative real‐time PCR (qRT‐PCR), in situ hybridization (ISH), immunohistochemistry, and western blot analysis, and it is localized in epithelial glands, perivascular regions, and the stromal compartment in cycling endometrium (Cervello et al., 2017; Gil‐sanchis et al., 2013; Krusche et al., 2007; Tempest et al., 2018). Studies have shown that LGR5^+^ cells from human endometrium have the ability to activate the stem cell niche (Cervello et al., 2017; Gargett et al., 2016; Tempest et al., 2018). Cervello et al. extracted LGR5^+^ cells from the human endometrium for subrenal transplantation and found that both LGR5^+^ and LGR5^–^ endometrial cells promote endometrial‐like cell regeneration (Cervello et al., 2017). LGR5^+^ endometrial cells not only express CD45 and CD163, but a transcriptome analysis suggested a putative hematopoietic origin. The LGR5^+^ macrophage‐like cells are possibly recruited from bone marrow and to activate the endometrial stem cell niche (Cervello et al., 2017). However, it was reported that two commercially available antibodies to the LGR5 protein showed nonspecific staining, casting considerable doubt on the hematopoietic origin of LGR5^+^ cells (Tempest et al., 2018). Thus, ISH and qRT‐PCR were used to demonstrate that LGR5 expressing cells are not limited to the postulated endometrial epithelial stem cell niche, but that LGR5 expression is regulated hormonally (Tempest et al., 2018). Therefore, we suggest that LGR5 is an important regulator for activating this stem cell niche, so regimens associated with niche regulation might be applied to promote adult stem cell function and endometrial regeneration, and potentially to treat refractory AS/EA.

## 6 PROGRESS AND CHALLENGES OF ENDOMETRIAL REPAIR BY ADULT STEM CELLS

**Figure 2:**
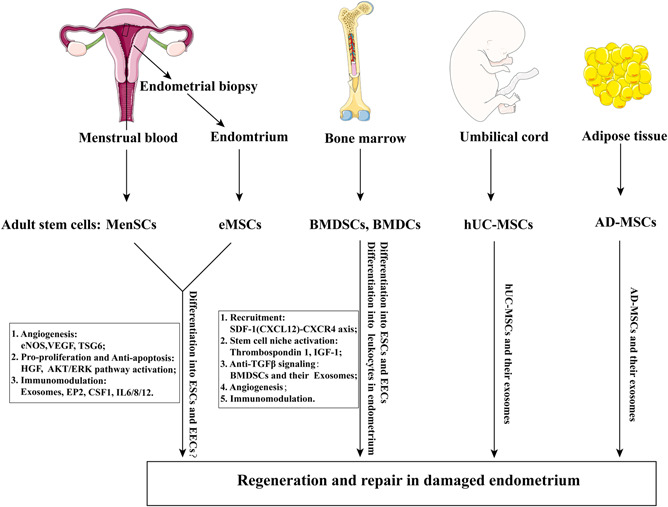
The roles of Adult stem cells for endometrial repair. A schematic diagram was established that how adult stem cells from endometrium, bone marrow, umbilical cord, and adipose tissue repair the damaged endometrium. MenSCs, eMSCs, BMDSCs, BMDCs, hUC‐MSCs, and AD‐MSCs act in regeneration of the damaged endometrium through direct differentiation or paracrine effects. These stem cells‐mediated paracrine factors, could participate in endometrial proliferation, angiogenesis, immunomodulation, and activate the stem cell niches to maintain stemness. There is no strong evidence that eMSCs or MenSCs differentiate into ESCs and EECs. Studies showed that BMDSCs and BMDCs can differentiate into ESCs and EECs. AD‐MSCs, adipose tissue‐derived mesenchymal stem cells; BMDCs, bone marrow‐derived cells; BMDSCs, bone marrow‐derived mesenchymal stem cells; CSF1, macrophage colony‐stimulating factor 1; EECs, endometrial epithelial cells; eMSCs, endometrial mesenchymal stem cells; eNOS, endothelial nitric oxide synthase; EP2, prostaglandin E receptor; ESCs, endometrial stromal cells; HGF, hepatocyte growth factor; hUC‐MSCs, human umbilical cord‐derived mesenchymal stem cells; IGF‐1, insulin‐like growth factor 1; MenSCs, menstrual blood‐derived stem cells; SDF‐1, stromal cell‐derived factor 1; TSG6, Tumor necrosis factor‐α‐induced protein 6; VEGF, vascular endothelial growth factor. The tissue pictures used in this figure derived from https://smart.servier.com/

A growing body of evidence supports an important role of adult stem cells in the regeneration and repair of the endometrium (Gargett et al., 2016). Therefore, clinical attempts have been made to use adult stem cells to treat AS/EA caused by dysfunction or excessive damage to the endometrial basalis layer. Here, we concentrate on current research progress in adult stem cell therapy and in the repair of injured endometrium as shown in Figure 2. It should be noted that there is no report of adult stem cell therapy for endometrial damage that occurs in cases of endometrial cancer. We proposed the potential mechanisms of adult stem cells to treat endometrial cancer are based on endometrial stromal cells' effects. It has been reported that conditioned medium (CM) from normal endometrial stromal cells in contact with Matrigel markedly reduced Ishikawa cell colony number and promote epithelial secretory product—glycodelin, when compared with CM from stromal cells cultured on plastic or unconditioned medium (Arnold et al., 2002). This suggests that normal endometrial stromal cells cultured in the appropriate extracellular matrix can modify the malignant phenotype of a well‐differentiated endometrial cancer cell line (Ishikawa). A study has proved the role of normal endometrial stromal cells‐mediated progesterone signaling in inhibiting endometrial carcinoma by epithelial‐stromal reconstruction model (Janzen, Cheng, et al., 2013). We supposes that normal endometrial stromal cells or their paracrine microenvironment with progesterone therapy may provide a means for a new fertility‐sparing strategy for patients with endometrial cancer in the future. Non‐clonogenic endometrial stromal cells and clonogenic perivascular eMSCs are significantly different cells. As eMSCs with double‐positive CD146 and PDGFRβ account for 1.5% of endometrial stromal cells and have multilineage differentiation (Schwab & Gargett, 2007), whether eMSCs, with or without progesterone therapy, can be used to treat endometrial carcinoma needs further study. On the other hand, we do not deny that the endometrial cancer‐derived stromal cells, called cancer‐associated fibroblasts, promote tumor cell proliferation when compared with stromal cells derived from normal endometrial tissues (Subramaniam et al., 2013). The crosstalk between normal stromal cells/MSCs and endometrial cancer cells should be paid attention to seriously.

### 6.1 Menstrual blood‐derived stem cells

Menstrual blood‐derived stem cells (MenSCs) are a novel source of menstrual fluid. Compared with other types of adult stem cells, MenSCs are easy to obtain in a noninvasive manner (Meng et al., 2007). They have a high proliferative capacity, short doubling time, multilineage differentiation potential, low immunogenicity, and low tumorigenicity, and maintain their karyotype even after 68 passages (Lv et al., 2018). Therefore, MenSCs can be used as ideal regenerative cells for the treatment of the female reproductive system, such as endometrial repair in patients with intrauterine adhesions, improvement of ovarian functions in those with premature ovarian failure, and repair of patients with pelvic organ prolapse. In addition, they can help in the treatment of myocardial infarction, stroke, liver injury, acute lung injury, and Duchenne muscular dystrophy through their differentiation and paracrine signals (Lv et al., 2018; Rossignoli et al., 2013), as well as exerting antitumor effects (e.g., cervical cancer, lung cancer, and neuroblastoma) to some extent (Chen, Qu, et al., 2019;  Moreno et al., 2017, 2019).

In 2007, Meng et al. first identified the MenSCs from menstrual blood (Meng et al., 2007). Human MenSCs express classical MSC markers (CD29, CD73, CD90, and CD105), and some other surface molecules (such as CD9, CD44, CD166, and OCT‐4), but do not express CD19, CD34, CD45, and CD133; they express human leukocyte antigen (HLA) ABC weakly, and do not express HLA‐DR, which suggests that they have low immunogenicity and a low rate of immune rejection (Khoury et al., 2014; Lv et al., 2018). MenSCs should not be considered as a kind of eMSCs as the two are different but relative cell types. The identity and definition of MenSCs should include the following three points (Chen, Qu, et al., 2019): (1) MenSCs should be derived from menstrual blood rather than the endometrial biopsies; (2) MenSCs express CD9, CD29, CD44, CD73, CD90, CD105, CD166, HLA‐ABC, and OCT‐4, while negative for the expression of CD19, CD34, CD45, CD133, and HLA‐DR; (3) MenSCs can be cultured and passaged in plastic‐adherent containers and display effective multilineage differentiation potential. However, the high‐quality and high‐consistency of MenSCs are still scarce because of the lack of a golden standardization and selectable molecular markers. If MenSCs are not purified or enriched with specific markers, they may be less consistent or effective than eMSCs derived from an endometrial biopsy (Darzi et al., 2016). Therefore, we believe that factors affecting the viability of MenSCs, such as the age of the donor, contraceptive use, and heterogeneous characteristics of stromal cells (Liu, Niu, et al., 2018; Lv et al., 2018), should be more considered in the future research.

In one study, autologous MenSCs were used to perform intrauterine transplantation after in vitro expansion to treat seven patients with severe uterine adhesions, of whom five patients had endometrial thickening up to 7 mm, two of four became successfully pregnant after embryo transfer, and one conceived naturally after the second autologous transplantation of MenSCs (Tan et al., 2016). Zheng et al. isolated and cultured OCT‐4^+^ MenSCs from patients with uterine adhesions and healthy volunteers and found, that the expression of CK and vimentin (VIM) in normal OCT‐4^+^ MenSCs cultured in the differentiation group using 17β‐estradiol and cytokines was significantly higher than that in the control group (Zheng et al., 2018). In the cultured MenSCs, 33.5 ± 1.5% cells were epithelial cell adhesion marker (EpCAM) positive. EpCAM is a molecule that expressed on the endometrial epithelia (Janzen, Cheng, et al., 2013), so the increased CK‐positive cells may be the proliferation of EpCAM‐positive epithelial cells under the action of estrogen, not necessarily related to the differentiation of MenSCs. Therefore, the evidence that CK‐positive endometrial epithelial cells differentiated from MenSCs is not strong. Because the colony‐forming ability of MenSCs and OCT‐4 expression in patients with severe intrauterine adhesions were impaired significantly, transplantation of MenSCs from healthy controls improved the status of intrauterine adhesions and increased the pregnancy rate (Zheng et al., 2018). In addition, extracellular vesicles collected from MenSCs participate in adaptive/innate immune responses, complement activation, antigen processing/presentation, and negative regulation of apoptosis, which can be used as immunomodulatory regulators for the treatment of inflammation‐related diseases (Marinaro et al., 2019). Mechanistically, MenSCs might induce angiogenesis‐related molecules, such as eNOS, VEGFA, VEGFR1, VEGFR2, and TIE2, by activating the AKT/ERK pathway to promote endometrial repair (Zhang et al., 2016). Besides, it has been demonstrated that MenSCs in spheroids increased transplanted cell survival and efficacy of stem cell therapy. Domnina et al. investigated that MenSCs in spheroids have the properties of MenSCs in a monolayer, such as specific CD molecule expression (CD73^+^, CD90^+^, CD105^+,^ CD140b^+^, CD45^−^, and CD34^−^), differentiation potential (adipocytes, osteoblasts, and decidual cells), a high proliferation rate and a high colony‐forming efficiency (Domnina et al., 2018; Zemelkoa et al., 2012). MenSCs in spheroids applied for murine AS model secreted significantly higher level of paracrine factors and promoted a higher pregnancy rate than monolayer MenSCs.

The complexity of the cellular components in MenSCs may bring uncertainties in clinical efficacy. Therefore, future studies should explore standard operating procedures for MenSCs, and focus on the safety and efficacy of clinical applications, duration of efficacy, and long‐term effects.

### 6.2 Endometrial mesenchymal stem cells

As mentioned above, eMSCs participate in endometrial regeneration and remodeling and are one of the most promising candidate adult stem cell types for stem cell therapy. They have functions of pro‐angiogenesis, antiapoptosis, immunomodulation, and chromatin stability maintenance without tumorigenicity and low immunogenicity after several passages of amplification (Gargett et al., 2016; Masuda et al., 2012; Schwab & Gargett, 2007). CD146^+^PDGFRβ^+^ endometrial perivascular cells have the functions and CD molecule expression characteristics of eMSCs (Schwab & Gargett, 2007). They highly express Cysteine angiogenic inducer 61 (CYR61) that plays an important role in angiogenesis in the damaged endometrium. Overexpression of CYR61 in endometrial perivascular CD146^+^PDGFRβ^+^ cells in combination with collagen scaffolds, can promote endometrial and myometrial regeneration in the damaged rat uterus, induce neovascularization and increase pregnancy rates (Li, Yan, et al., 2019). In addition, extracellular vesicles collected from eMSCs participate in adaptive/innate immune responses, complement activation, antigen processing/presentation, and negative regulation of apoptosis, which can be used as immunomodulatory regulators for the treatment of inflammation‐related diseases (Marinaro et al., 2019).

Tersoglio et al. conducted a longitudinal and in‐depth pilot study in which infertile women who had failed repeated embryo transplantation underwent subendometrial transplantation of eMSCs. The endometrium thickened from 5.2 ± 1.24 mm before treatment to 9.93± 0.77 mm after (*p* = 0.000) and the clinical pregnancy rate reached 79.31% (23/29) (Tersoglio et al., 2020). Furthermore, the live birth rate following embryo transfer was 45.45% (10/22), and the rate of continued pregnancy was 24.14% (7/29). This suggests that subendometrial transplantation of eMSCs can significantly increase the thickness of the endometrium and greatly improves in vitro fertilization outcomes. In addition, eMSCs differentiate into mesodermal and ectodermal cell lineages such as insulin‐secreting islet cells that have potential application value in treating diabetes (Santamaria et al., 2011). Furthermore, the PI3K/AKT signaling pathway inhibitor LY294002 promoted the differentiation of eMSCs on polycaprolactone/collagen scaffolds into motor neurons (Ebrahimi‐Barough et al., 2017).

Senescence is one of the major reasons for stem cell therapy failure. Oxidative stress in the extracellular environment initiates the senescence‐associated secretory phenotype and changes the paracrine characteristics of eMSCs (Burova et al., 2013), which may be the reason for the high cell mortality rate and difficulty in amplifying eMSCs after transplantation. Cho et al. (2019) discovered an endogenous anti‐senescence factor, Sonic Hedgehog (SHH). SHH is a morphogenesis‐inducing factor during embryonic development, and SHH expression and activity in stem cells are reduced significantly with aging. Exogenous application of SHH effectively alleviates various aging‐associated declines in endometrial stem cell functions including proliferation, migration, and senescence‐associated β‐galactosidase enzymatic activity. Telomere shortening is an important mechanism that drives replicative senescence (de Magalhaes & Passos, 2018). Additionally, increasing evidence supports a role of oxidative stress in induction of senescence. It is reported that insulin‐like growth factor binding protein 3 (IGFBP3) induces apoptosis in an IGF‐1‐dependent or ‐independent manner (Huang et al., 2003). Vassilieva et al. found that premature senescence of eMSCs induced by oxidative stress promoted the secretion of IGFBP3 into culture medium. After neutralizing IGFBP3 in the culture medium, the effect of IGFBP3 on inducing senescence of young eMSCs was obviously weakened, and the ability to promote proliferation was enhanced. The application of synthetic IGFBP3 also induces senescence of eMSCs and reduces the expression of stem gene CD146/MCAM in eMSCs (Vassilieva et al., 2020).

The differentiation direction of eMSCs is extremely susceptible to the microenvironment. Zhang et al. found that peritoneal washes from patients with endometriosis promoted the differentiation of eMSCs into myofibroblasts, leading to adhesions, anatomical abnormalities, and pelvic pain (Zhang et al., 2019). Reshaping the stem cell microenvironment to avoid abnormal differentiation of eMSCs is a difficult problem that needs to be overcome.

### 6.3 Bone marrow‐derived stem cells and bone marrow‐derived cells

In 2004, it was reported that donor‐derived endometrial cells were detected in endometrial samples from bone marrow transplant recipients (Taylor, 2004). In endometrial samples from patients with leukemia hematologic tumors who underwent bone marrow transplantation, donor‐derived endometrium was detected with a mismatched HLA type, accounting for 0.2%–48% and 0.3%–52% of the endometrial epithelium and stroma, respectively. Ikoma et al. also found Y‐chromosome‐positive endometrial cells in the endometrium of female patients with hematological tumors who underwent male bone marrow transplantation, of which Y‐positive glandular epithelial and stromal cells accounted for 0.6%–8.4% and 8.2%–9.8%, respectively (Ikoma et al., 2009). Moreover, murine experiments have also confirmed that bone marrow‐derived stem cells (BMDSCs) can differentiated into endometrial cells. Alawadhi et al. found that Y^+^CD45^‐^ cells in an endometrial injury group were two‐fold higher than those in the uninjured group after tail vein injection of male C57BL/6 BMDSCs (Alawadhi et al., 2014). Y^+^CD45^‐^CK^+^ cells and Y^+^CD45^‐^CK^‐^ cells demonstrated BM‐derived epithelial and stromal cells in the endometrium respectively. And the transplantation group also had a higher pregnancy rate compared with the non‐transplanted group (90% vs. 30%, *p* = 0.0225) (Alawadhi et al., 2014). Similarly, Cervello and collegues reported that human CD133^+^ BMDSCs, with intrauterine or tail vein injection in a murine model of EA, could engraft around the endometrial vessels and promote endometrial regeneration through paracrine actions (Cervello et al., 2015; de Miguel‐Gomez et al., 2020). These findings suggest that endometrial cells can be differentiated from donor BM cells, that is, stem cells from non‐endometrial sources can also participate in endometrial tissue regeneration. But the identity of the stem cells from the bone marrow has not actually been determined. On the contrary, Ong et al. failed to observe the transdifferentiation capacity of BM stem cells in chimeric mouse models (Ong et al., 2018). They found that macrophages weakly expressing CD45 were abundant in the stroma, infiltrated the epithelial and vascular compartments, and could be easily be mistaken for BM‐derived endometrial cells.

Increasing evidence shows that endometrial regeneration also depends, at least partly, on recruitment and engraftment of bone marrow–derived cells (BMDCs) and their subsequent differentiation into nonhematopoietic endometrial cells. Gil‐Sanchis and collegues reported a comparative study between four freshly isolated and three culture isolated murine BMDC populations in endometrial regeneration in mice after nonlethal irradiation (Gil‐Sanchis et al., 2015). They found that freshly isolated MSCs and endothelial progenitor cells together with BMDC hypoblast‐like stem cells induced the greatest degree of regeneration, whereas culture isolated MSCs and OCT‐4 negative BMDC multipotent adult progenitor cell transplantation may have an opposite effect on endometrial regeneration. The different behaviors between freshly isolated MSCs and in vitro cultured MSCs can also be explained by the loss of surface receptors (e.g., CXCR4) during cell culture, which is important for the homing of cells to damaged tissues. Moreover, BMDCs were detectable as early as 3 months after bone marrow transplantation, and the BM remained a long‐term contributor of nonhematopoietic endometrial cells (Morelli et al., 2013). These findings suggest that BMDCs may serve as an important contributor of stem cells to the endometrium, and may potentially restore endometrial fertility after irradiation for oncological indications. Besides, BMDCs have a previously unrecognized nonhematopoietic physiologic contribution to decidual stroma, thereby playing important roles in blastocyst implantation and pregnancy maintenance. Pregnancy mobilizes BMSCs to the circulation and induces considerable adult BMDCs recruitment to decidua, where some differentiate into nonhematopoietic decidual cells that express prolactin (Tal et al., 2019). In addition, BMDCs had more advantages in stem cell amplification than uterus‐derived cells, and recruitment of BMDCs to the endometrium was higher when injected intravenously via the tail compared with intrauterine injection (Liu, Tal, et al., 2018). All these findings suggests the important roles of BMDCs in endometrial fertility. However, the identity of which cells in the BMDCs contribute to endometrial regeneration requires more in‐depth research.

Besides, studies demenstrated that bone marrow mesenchymal stem cells (BMSCs) have great potential in repairing damaged endometrium and recovering endometrial function. After treatment with BMSCs, the endometrial tissue in a murine model of EA showed a significantly thicker lining, with increased levels of anti‐inflammatory cytokines (such as FGF and IL‐6) and decreased pro‐inflammatory cytokines (such as TNF‐α and IL‐1β) (Zhao et al., 2015). Moreover, electroacupuncture and tissue engineering materials such as collagen scaffolds can enhance the effect of BMSCs on repairing injured endometrium (Ding et al., 2014; Xia et al., 2019). It has been reported that migration of BMSCs to the endometrium and stem cell differentiation are promoted by ischemia/reperfusion injury and are inhibited by cigarette smoke exposure (Du et al., 2012; Zhou et al., 2011). Exosomes from BMSCs also participate in the repair of damaged endometrium. TGFβ is a major regulator that drives endometrial injury and promotes fibrous tissue formation (Salma et al., 2016). Exogenous application of TGFβ induced endometrial epithelial‐mesenchymal transition and increased the apoptotic level of endometrial epithelium (Yao et al., 2019). Exosomes from BMSCs reversed the TGFβ‐mediated epithelial–mesenchymal transition, promoted epithelial cell proliferation and reduced the fibrous tissue area. However, it is unclear which components of BMSC‐derived exosomes can repair damaged endometrium.

The CXCL12‐CXCR4 axis was shown to play an important role in recruiting BMDCs to the local endometrium and in promoting endometrial repair by cell‐labeling experiments. Yi et al. (2019) found that CXCL12 and BMDCs acted synergistically in increasing the endometrial thickness of the damaged endometrium, and in improving pregnancy rates and litter sizes.

Furthermore, a randomized, double‐blind, placebo‐controlled clinical trial evaluated the effects of DDP4 inhibitors in regulating the endometrial decidual cells in recurrent miscarriage; DDP4 inhibitors mainly increase CXCL12 bioactivity, which at least partly depends on recruitment and engraftment of BMDCs and their subsequent differentiation into nonhematopoietic endometrial lineage, thereby increasing the mid‐luteal phase endometrial CFUs, endometrial thickness, and 12‐month pregnancy outcomes in women with a history of recurrent miscarriage (Tewary et al., 2020). They found that the mid‐luteal endometrial CFU number in the DDP4 inhibitor group was obviously increased and DIO2 expression from senescent decidual cells was significantly decreased. Besides, there was a synergistic effect of electroacupuncture and bone marrow mesenchymal stem cells (BMSCs) in repairing the damaged endometrium of Sprague‐Dawley rats. The potential mechanism is that electroacupuncture activates the CXCL12‐CXCR4 axis to promote BM stem cell recruitment to the endometrium and enhances their paracrine signaling (Xia et al., 2019). These findings suggested the clinical application potential of DDP4 inhibitor in repairing damaged endometrium and improving pregnancy outcomes.

It was reported that BMDCs could differentiate into nonhematopoietic endometrial epithelial and stromal cells. However, BMDCs could also be recruited to endometriotic foci, supporting the theory of their involvement in the pathogenesis of endometriosis (Du & Taylor, 2007). Avoiding the recruitment of BMDCs to sites of endometriosis is an issue that needs to be overcome.

### 6.4 Umbilical cord‐derived mesenchymal stem cells

Human umbilical cord‐derived mesenchymal stem cells (hUC‐MSCs) are widely used in tissue damage repair including applications to damaged myocardium, liver, lung, kidney, skin mucous membrane and vascular endothelium, endometrium, and ovarian tissue (Xin et al., 2019; Xu et al., 2017). A team from Nanjing Drum Tower Hospital in China conducted a clinical trial by combined application of hUC‐MSCs and a degradable collagen scaffold for treating patients with intrauterine adhesions (Cao et al., 2018). This regimen enhanced the repair ability of the endometrium and alleviated intrauterine adhesions. A study has also shown that hUC‐MSC exosomes promoted the proliferation of allogeneic endometrial stroma and endometrial repair (Lv et al., 2020), suggesting the importance of paracrine microenvironment of hUC‐MSCs. Although the specific mechanisms involved are not clear, we suggest that endometrial stromal cells differentiated from MSCs play important roles in response to endometrial injury. Endometrial stromal cell proliferation and mesenchymal–epithelial transition might be responsible for the mechanisms underlying the MSCs involved in endometrial regeneration (Owusu‐Akyaw et al., 2019; Patterson et al., 2013; Yin et al., 2019). On the other hand, extracellular vesicles derived from hUC‐MSCs deliver miR‐302a to the local area of endometrial cancer, inhibit the signals of cyclin D1 and AKT1, and prevent the progression of endometrial cancer (Li, Liu, et al., 2019). These studies are not contradictory, suggesting that hUC‐MSCs can be one of fertility‐sparing therapies for endometrial cancer in the future by not only inhibiting the growth of endometrial cancer cells directly, but also repairing the damaged endometrium after tumor complete remission.

### 6.5 Adipose tissue‐derived mesenchymal stem cells

Adipose tissue‐derived mesenchymal stem cells (AD‐MSCs) have the advantages of large reserves in vivo, they are easy to obtain and have the strong proliferative capacity, and their applications in the tissue engineering field have gradually shown great potential, including the treatment of diabetes, osteoarthritis, and nerve injury repair (Bydon et al., 2020; Lee et al., 2019; Liu et al., 2016). There are relatively few studies on AD‐MSCs in endometrial repair. AD‐MSCs and their exosomes promote regeneration of the endometrium in rats with intrauterine adhesion, improve endometrial receptivity, and reshape endometrium fertility (Shao et al., 2019; Zhao et al., 2020), which indicates the local application of AD‐MSC exosomes in the uterus may be a promising treatment for patients with intrauterine adhesion. Other studies have shown that conditioned medium from endometrial cancer cell‐pretreated adipose‐derived stem cells (ADSCs) promotes endometrial cancer cell proliferation and migration by activating the STAT3 signaling pathway (Chu et al., 2018), which suggests that ADSCs are context‐dependent by the tumor milieu.

### 6.6 Other tissue‐derived stem cells

#### 6.6.1 Human amniotic epithelial cells

These have the characteristics of stem cells. After intraperitoneal injection of human amniotic epithelial cells, the endometrial thickness, the number of glands, and number of microvessels in mice with uterine adhesion were increased significantly, the fibrous tissue area was reduced, and the pregnancy rate was improved greatly (Li, Zhang, et al., 2019).

#### 6.6.2 Oral mucosal epithelial cells

These have a high potential for proliferation in vitro and can be used as seed cells in tissue engineering. They can grow on a decellularized and lyophilized amniotic membrane (DL‐AM), and effectively reduce the degree of intrauterine adhesion in mice, and restore endometrial fertility (Chen, Sun, et al., 2019). How oral mucosal epithelial cells carried by DL‐AM improve regeneration of the endometrium is unclear and needs further study.

#### 6.6.3 Human induced pluripotent stem (hiPS) cells

It was found that human induced pluripotent stem (hiPS) cells cultured in vitro gradually differentiate into intermediate mesoderm, coelomic epithelium, and the Müllerian duct, and eventually produce endometrial stromal fibroblasts by a sequential series of regulatory factors sequentially. In this process, the WNT/CTNNB1 signaling pathway plays important roles (Miyazaki et al., 2018). These findings suggest that hiPS cells may be used for cell therapy of endometrium‐related diseases such as AS, endometriosis, early endometrial cancer, and uterine‐associated infertility. However, because this review is mainly about the application of adult stem cells for endometrial repair, so hiPS cells are not discussed in more detail here.

In summary, adult stem cells derived from menstrual blood, endometrium, bone marrow, and umbilical cord act in regeneration of the damaged endometrium through direct differentiation or paracrine effects. These stem cells‐mediated paracrine factors, could participate in endometrial proliferation, angiogenesis, immunomodulation, and activate the stem cell niches to maintain stemness as shown in Figure 2.

## 7 TISSUE ENGINEERING MATERIALS

One potential factor affecting the efficacy of adult stem cell therapy in repairing organs is the difficulty of long‐term engraftment of transplanted adult stem cells to target tissues (Dimmeler et al., 2014). Studies have shown that in the first few days after transplantation, the percentage of transplanted stem cells surviving in the injury tissue is quite low, and there is almost no survival even after 3 months. Some microenvironmental factors such as hypoxia and nutritional deficiency might disturb the viability of transplanted stem cells in the injured tissue (Potier et al., 2007). Thus, we believe that biomaterials may be a potential solution to improve stem cell viability. Here we list several biomaterials currently used in the treatment of AS in animal models and in humans.

### 7.1 Hyaluronic‐acid gel (HA‐GEL)

Hyaluronic‐acid gel (HA‐GEL) has been approved for clinical intrauterine injection to prevent postoperative intrauterine adhesions by the China Food and Drug Administration. Meanwhile, HA‐GEL has shown satifactory biocompatibility when carrying a variety of cells in cell therapy, such as AD‐MSCs, hUC‐MSCs, and even functionalized endometrial stromal cells (Feng et al., 2017; Kim et al., 2019; Wang et al., 2020). HA‐GEL/hUC‐MSCs complex transplantation significantly promoted endometrial regeneration in rhesus monkeys with severe endometrial injury, characterized by an increase in the number of glands and in endometrial thickness (Wang et al., 2020). In addition a sustained release system by using crosslinked HA as an agent for the sustained release of MSC‐secretome could effectively repair endometrial injury in rats (Liu, Hu, et al., 2019). These findings suggested that HA‐GEL can improve the viability of MSCs and the effects of endometrial repair.

### 7.2 Collagen scaffold

Degradable collagen scaffold with pores of 20–200 μm in diameter was able to provide a three‐dimensional scaffold for cell proliferation, differentiation, and infiltration. In 2018, a Phase I clinical trial proved the safety and efficacy of transplanting clinical‐grade hUC‐MSCs loaded on collagen scaffold into the uterine cavity for patients with recurrent intrauterine adhesions (Cao et al., 2018). Besides, collagen scaffolds implanted with BMSCs could not only maintain the stemness of transplanted BMSCs in the injured endometrium, but also effectively promote regeneration of the damaged endometrium, increase the endometrial thickness, reduce fibrous scar formation, and improve the pregnancy and live birth rates (Ding et al., 2014). The use of collagen scaffolds significantly improved the efficacy of therapeutic stem cells by maintaining their viability and extending the duration of contact with the damaged endometrium (Xu et al., 2017).

### 7.3 Hydrogels

Synthetic hydrogels deserve attention because their mechanical properties and cell‐binding capability can be controlled. Yao et al. designed a nanocomposite aloe/poloxamer hydrogel for β‐estradiol intrauterine delivery and in situ administration showed excellent endometrial regeneration in the treatment of patients with AS (Yao et al., 2020). Therefore, intelligently manipulating tissue engineering materials for endometrial repair of adult stem cells is likely to be the focus of future research.

## 8 DISCUSSION AND FUTURE PERSPECTIVES

Currently, stem cells derived from various tissues, such as enMSCs, eMSCs, BMSCs, and UC‐MSCs, have shown great beneficial effects on the repair of the injured endometrium. Furthermore, tissue engineering with scaffold materials might effectively improve the repair efficacy of stem cells in the damaged endometrium by maintaining stem cell viability and function. Some combinatorial treatments with stem cells and tissue engineering scaffold materials have entered the clinical trials and have demonstrated excellent endometrial repair (Cao et al., 2018). Successful application of these stem cells to the treatment of endometrial atrophy, extensive intrauterine adhesions, and scarring will improve menstruation and help enhance female fertility.

However, there remains a lack of research on the repair of the damaged endometrium after reversal of a gynecological tumors at present, especially for endometrial cancer. During fertility‐preserving therapy, frequent intrauterine operations including dilation and curettage and endometrial biopsy, can cause endometrial scarring repair and intrauterine adhesions. According to reports, the pregnancy rates among these patients is still very low: 41.2% in patients with atypical hyperplasia and 34.8% in those with endometrial cancer, not to mention the live birth rate (Gunderson et al., 2012). Inoue and collegues evaluated the factors that affected the establishment of clinical pregnancies by comparing a pregnancy group with a nonpregnant group of patients treated with medroxyprogesterone acetate (Inoue et al., 2016). They determined recurrence, endometrial thickness during ovulation and age as three factors affecting pregnancy establishment following conservative treatment (Inoue et al., 2016). Due to the lack of safe and effective endometrial repair strategies, moderate or severe intrauterine adhesions, or thin endometrium cannot effectively support embryo implantation. The current clinical treatment of infertility caused by endometrial damage is high‐dose estrogen therapy (Evans et al., 2016). However, this treatment plan can induce the recurrence of endometrial cancer while repairing the endometrium. Therefore, there is an urgent need to develop safe and effective techniques to successfully repair the damaged endometrium and improve the reproductive outcomes without causing cancer recurrence.

Various reports have shown that MSCs can inhibit tumor growth and angiogenesis by secreting exosomes, paracrine factors, and by regulating the local immune environment, thereby inhibiting tumor progression (Qiao et al., 2008; Sun et al., 2009). For example, human umbilical cord and fat‐derived MSCs were found to inhibit lung growth and metastases of transplanted breast cancers by increasing poly (ADP‐ribose) polymerase‐1 (PARP) cleavage and caspase‐3 expression (Sun et al., 2009). In addition, conditioned media derived from Z3 human MSC cultures showed decreased colony‐forming ability and decreased proliferation by inhibiting Wnt signaling in the human hepatoma cell lines H7402 and HepG2 (Qiao et al., 2008). However, there is controversy regarding the role of adult stem cells in tumor growth and progression. It has been reported that omental adipose stromal cells, a multipotent population of MSCs contained in the omental tissue, can promote tumor growth and induce therapy resistance by upregulating glycolysis and reducing oxidative stress in endometrial and ovarian cancer cells (Salimian et al., 2015). A number of basic studies and clinical trials have tried to use MSCs transplanted from different tissues (umbilical cord, fat, bone marrow, etc.) to treat AS/EA, which showed no tumorigenicity (Cao et al., 2018). However, there remains a lack of research on the repair of the damaged endometrium after reversal of endometrial cancer.

These adult stem cells might be an important research direction for repairing damaged endometrium after endometrial cancer remission. We believe that ensuring the safety and effectiveness of this approach will be the key. Normal MSCs are converted by some inflammatory factors such as TNF‐α into tumor‐associated MSCs via CCR2‐dependent recruitment of tumor‐promoting macrophages (Ren et al., 2012). This suggests that MSCs are easily affected by the inflammatory microenvironment, or more inclined to be converted to tumor‐associated MSCs through reprogramming secretion profiles. Therefore, blocking the signaling pathways of MSCs “education” in inflammatory microenvironment might effectively prevent MSCs from being converted to tumor‐associated MSCs, thereby ensuring the safety of their application. In this regard, other factors such as IGFBP3, which induces oxidative stress, can drive adult stem cell senescence through inhibiting stem cell differentiation and expansion (Vassilieva et al., 2020). Eliminating or blocking the molecules that promote senescence of MSCs or introducing tissue engineering scaffold materials into MSCs therapeutic systems might effectively maintain stem cell differentiation and secretion spectrum, thereby increasing the effectiveness of adult stem cell therapy. Besides, MSCs are heterogeneous cell populations (Phinney, 2012). Finding a certain subtype among these MSCs, that can not only effectively repair the damaged endometrium, but also avoid the recurrence of endometrial cancer, will be a precision treatment strategy needed for recovering normal endometrial function after endometrial cancer remission. Therefore, we believe that the problems or challenges that arise during MSC therapies will help inspire new solutions for this.

## CONFLICT OF INTERESTS

All the authors declare that there are no conflicts of interests.

## Data Availability

Data sharing not applicable to this article as no datasets were generated or analyzed during the current study.
